# An Evaluation of Food Insecurity and Its Correlates in a Filipino American Study Sample Residing in Clark County, Nevada

**DOI:** 10.1089/heq.2019.0074

**Published:** 2019-10-24

**Authors:** Prescott Cheong, Courtney Coughenour, Marya Shegog, Saruna Ghimire, Lawrence Sagadraca, Francisco Sy

**Affiliations:** ^1^ School of Public Health, University of Nevada, Las Vegas, Las Vegas, Nevada.; ^2^ The Lincy Institute, University of Nevada, Las Vegas, Las Vegas, Nevada.; ^3^ Department of Sociology and Gerontology, Miami University, Oxford, Ohio.

**Keywords:** Asian American, food security, Filipino American, public health, Social Ecological Model, Southern Nevada

## Abstract

**Purpose:** Filipino Americans comprise over half of the Asian American population in Clark County, Nevada. Despite their large numbers, food insecurity rates are aggregated with the entire Asian American population. In 2016, 1.6% of Asian American households in Clark County were food insecure, yet, 22% of households reported annual incomes at or below 200% of the federal poverty level. This study aimed to assess the status and correlates of food insecurity specific to Filipino Americans in Clark County, Nevada.

**Methods:** The United States Department of Agriculture (USDA) Short Form Food Security Module was administered among 192 Filipino Americans residing in Clark County, NV. Results were viewed through the theoretical framework of the Social Ecological Model.

**Results:** 27.1% of respondents experienced food insecurity within the past year. Adjusted logistic regression revealed that incomes less than $20,000 (odds ratio [OR]=4.13, 95% confidence interval [CI]: 1.43–11.9), having no health insurance (OR=5.22, 95% CI: 1.67–16.34), and eating mainly American or Western foods (OR=7.3, 95% CI: 1.73–30.77) were significant predictors of food insecurity.

**Conclusions:** A significantly higher prevalence of food insecurity among Filipino American subpopulations, compared to the estimates for Asian Americans in Clark County, suggests the need to disaggregate data for Asian American subgroups. The Social Ecological Model provides greater context to the findings identifying that the intrapersonal and policy level factors were associated with food insecurity among our participants, thus suggesting the need to utilize multilevel interventions to address food insecurity in Filipino Americans. The findings may be utilized to inform future interventions aimed at improving the overall health and food security among Filipino Americans.

## Introduction

The United States Department of Agriculture (USDA) defines food insecurity as “a household-level economic and social condition of limited or uncertain access to adequate food.”^1^ In 2016, ∼12.3% of all households in the United States were food insecure.^1,2^ Food insecurity is associated with many health consequences such as increased rates of chronic disease and health risk factors such as diabetes,^3^ hypertension,^3,4^ nutritional deficiencies,^5^ and a higher likelihood of reporting fair/poor health.^5^ As such, numerous public and private enterprises continue to invest efforts toward improving and maintaining food security in the United States.

Certain households are disproportionately affected by food insecurity. In particular, low-income households, households headed by ethnic minorities, households with children headed by single women, and rural households are more vulnerable to food insecurity. Regional differences exist, with Southern states reporting higher food insecurity.^2^ Research examining food insecurity among the Asian American population is limited and is especially lacking among Filipino Americans. In general, Asian Americans are among the most understudied ethnic minority groups.^6–8^ Stereotypes that purport Asian Americans as a self-sufficient, well-educated, and upwardly mobile group may contribute to this limited body of research.^6^ Furthermore, despite having a diverse subpopulation, the available health data on Asian Americans are often aggregated, thus inhibiting inferences at the subgroup level.^9^ Data aggregation masks the health needs of the most vulnerable groups in the Asian American community.^10^

A study analyzing food insecurity rates among California residents from 2001 to 2011 found that U.S.-born Asians had similar food insecurity rates to U.S.-born whites.^11^ The same study noted that naturalized or legal permanent resident Asian immigrants recorded greater food insecurity rates than U.S.-born whites.^11^ Studies focusing specifically on Filipino Americans suggest that certain health outcomes and behaviors such as hypertension, type 2 diabetes, smoking, and alcohol use are worse in Filipino Americans compared with other racial groups and Asian American subgroups.^8,12^ A previous study using the California Health Interview Survey documented that the prevalence of food insecurity rates differ considerably between Asian American subgroups, with the highest prevalence among Vietnamese (16.42%) and the lowest prevalence among Japanese (2.28%); the prevalence rate for Filipino Americans was 8.26%.^13^ Nonetheless, the limited literature available on Filipino American food insecurity indicates that this topic warrants further investigation.^14,15^

Filipino Americans represent one of the largest and most diverse immigrant populations,^12^ with nearly 3.4 million currently residing in the United States.^16^ Estimates rank Filipino Americans as the second most populous Asian subgroup, behind Chinese Americans.^17^ In Clark County, Nevada, Filipino Americans comprise over half of the Asian American population or ∼5% of the total population.^18^ Although Filipino Americans make up a significant portion of the U.S. population, their particular health issues remain poorly understood.^8^ Prominent Asian American researchers have called for increased visibility of Filipino American health issues and to utilize a comprehensive strategy to view these issues and ultimately intervene in an appropriate manner.^8^

Food insecurity data specific to Filipino Americans in Clark County are unavailable due to aggregation with other Asian American subgroups. The reported Asian American food insecurity rate in 2016 for Clark County, Nevada was 1.6%, which is considerably lower than the food insecurity rate for non-Hispanic whites at 15.5%.^19^ Yet, this is seemingly inconsistent, as 24% of Filipinos report household incomes of 200% below the federal poverty level compared to 33% of whites.^20^ These limitations and inconsistencies provide an opportunity to research this previously under studied, yet, important area. Thus, the purpose of this study is to assess the status and correlates food insecurity among Filipino Americans residing in Clark County, Nevada. This is one of the first studies to specifically examine food insecurity in an exclusively Filipino American sample. The Social Ecological Model conceived by McLeroy et al.^21^ provided the theoretical framework to identify the salient sociocultural and environmental factors that influenced food insecurity.

## Methods

### Study design and participant recruitment

This study is a part of a larger comprehensive health needs assessment of Filipino Americans in Clark County, Nevada.^22^ This cross-sectional study included 200 Filipino Americans, aged 18+ years, residing in Clark County, Nevada. Convenience sampling recruited participants at Filipino ethnic association events, organizations' meetings, church events, and a Filipino grocery store. Participants were given the option of completing the self-administered paper survey in person, by mail, or online. Data were collected between April and August 2017. Although the survey was available in Tagalog, all participants elected to complete the survey in English. Study protocol was approved by UNLV Institutional Review Board.

### Study variables

#### Food security

Food security status was the main outcome (dependent variable) of the study, and was determined using the “U.S. Household Food Security Survey Module: Six-Item Short Form.”^23^ The short form is a reliable and valid instrument with good overall concordance, high sensitivity (92.0%), and high specificity (99.4%).^24^ The Cronbach's alpha of the tool in this study was 0.64. Food security status was calculated as high or marginal food security (raw score 0–1), low food security (raw score 2–4), and very low food security (raw score 5–6). In the final analysis, food security status was dichotomized to food secure (raw score 0–1) and food insecure (raw score 2–6).^23^

### Social ecological model and food security correlates

Utilizing the levels of McLeroy's Social Ecological Model,^21^ variables in the intrapersonal level included age, body mass index (BMI), annual household income, employment status, educational level, self-reported chronic disease status (diabetes, hypertension, high cholesterol), years lived in the United States, fast food consumption frequency, and usual type of food eaten (i.e., Filipino or Western/American); interpersonal level included children younger than the age of 18 years in the household; community level included geographic location by city jurisdiction; and policy level included type of health insurance coverage.

The details of sociodemographic variables collected in the Filipino health needs assessment have been previously reported.^22^ In brief, the survey tool contained questions regarding demographics, acculturative status, health behaviors, health conditions, access and barriers to health care, and perceived Filipino community health needs. All measures, including household income, participants' height and weight for BMI calculation, and disease status, were self-reported. The tool was previously pretested among Filipino Americans in Philadelphia to ensure scientific and cultural appropriateness and underwent multiple revisions as a result of feedback during pilot testing.^12^

### Statistical analyses

Statistical analyses were performed in SPSS™ Version 24. Differences in mean values and frequency distributions between food secure and insecure households were assessed using independent *t*-tests and Pearson's chi-square test (χ^2^), respectively. A chi-square goodness of fit test was conducted to compare the prevalence of food insecurity in the study population with the 2016 food insecurity rate among “Asians Only” in Clark County attained from the Food Security Supplement to the U.S. Census Bureau's Current Population Survey (CPS).^19^ In the absence of disaggregated estimates, we used the food insecurity prevalence available for the “Asian Americans” as a proxy for Filipino Americans.

Univariate and multivariable binary logistic regression was conducted to ascertain significant correlates of food insecurity. Model 1 was adjusted for all independent predictors that were statistically significant in the univariate modeling. Model 2 included all variables of interest, regardless of statistical significance in the univariate modeling.

## Results

### Study population characteristics

The general demographics of the study population, overall and by food security status, are summarized in Table 1. The majority of respondents were female (65%), with a mean age of 49.4 years (±18.1), employed (62%), had attended or completed some form of college education (82%), and were from households with more than $40,000 annual income (54%). Approximately 82% of respondents were born outside the U.S. but had lived in the United States for almost 25 years on average. The majority (69%) of the respondents usually ate both Filipino and Western/American food, while 9.2% of respondents indicated that they usually ate Western/American food. Self-reported health variables showed high rates of hypertension (47%), high cholesterol (46%), and diabetes (23.8%). The mean BMI was determined to be 25.3 kg/m^2^ (±6.6). The majority of respondents possessed some form of health insurance (89%).

**Table 1.** Demographic and Socioeconomic Characteristics of a Subsample of Filipino Americans in Clark County, Nevada by Food Security Status and Statistical Analysis of Differences Between Groups

Socio-demographic variables		Food security status		
	Total sample	Food secure	Food insecure	
	*n*=200	*n*=140 (72.9%)	*n*=52 (27.1%)	
	*n* (%)	*n* (%)	*n* (%)	*p*
Age, years (mean±SD)	49.4±18.1	50.4±17.7	47.6±19.2	0.334^a^
Gender				0.843
Male	70 (35.0)	49 (35)	19 (36.5)	
Female	130 (65.0)	91 (65)	33 (63.5)	
Educational status				0.111
High school or below	36 (18.3)	21 (15)	14 (27.5)	
College or associate	109 (55.3)	79 (56.4)	27 (52.9)	
Graduate and above	52 (26.4)	40 (28.6)	10 (19.6)	
Employment status				0.383
Employed	123 (62.1)	89 (63.6)	28 (54.9)	
Unemployed	21 (10.6)	13 (9.3)	8 (15.7)	
Retired	54 (27.3)	38 (27.1)	15 (29.4)	
Annual household income				**0.001**
Less than $20,000	40 (21.1)	22 (16.2)	18 (37.5)	
$20,000–$40,000	48 (25.3)	30 (22.1)	15 (31.3)	
Above $40,000	102 (53.7)	84 (61.8)	15 (31.3)	
Household composition				0.237
HH with children under 18	43 (24.3)	34 (26.6)	8 (17.8)	
HH without children under 18	134 (75.7)	94 (73.4)	37 (82.2)	
Residence by city boundaries				0.341
Henderson	25 (12.8)	18 (13)	8 (15.7)	
City of Las Vegas	59 (30.3)	46 (33.3)	11 (21.6)	
North Las Vegas	11 (5.6)	9 (6.5)	2 (3.9)	
Clark County	100 (51.3)	65 (47.1)	30 (58.8)	
Years resided in the U.S.				
Mean±SD	24.9±13.9	25.4±14.6	23.7±12.7	0.454^a^
Resided in U.S. for 0–10 years	37 (20.2)	27 (21.1)	9 (18.4)	0.373
Resided in U.S. for 11–25 years	67 (36.6)	43 (33.6)	22 (44.9)	
Resided in U.S. for ≥25 years	79 (43.2)	58 (45.3)	18 (36.7)	
Usual type of food eaten				0.077
Filipino food	42 (21.5)	32 (23)	9 (18)	
Western or American food	18 (9.2)	8 (5.8)	8 (16)	
Both	135 (69.2)	99 (71.2)	33 (66)	
BMI, kg/m^2^ (mean±SD)	25.3±6.6	25.5±5.9	24.9±7.9	0.619^a^
Self-reported diabetes status				0.169
Yes	46 (23.8)	28 (20.4)	15 (30)	
No	147 (7.2)	109 (79.6)	35 (70)	
Self-reported hypertension status				
Yes	92 (46.9)	61 (44.2)	28 (53.8)	0.235
No	104 (53.1)	77 (55.8)	24 (46.2)	
Self-reported high cholesterol status				0.413
Yes	91 (46)	67 (47.9)	21 (41.2)	
No	107 (54)	73 (52.1)	30 (58.8)	
Type of health insurance				**0.007**
Private insurance	102 (53)	75 (55.1)	20 (38.5)	
Public insurance	70 (36)	51 (37.5)	20 (38.5)	
No insurance	22 (11)	10 (7.4)	12 (23.1)	

^a^ *p*-value from independent *t*-test; others are from chi-square test.

Bold values indicate statistical significance, *p*≤0.05.

BMI, body mass index; SD, standard deviation; U.S., United States.

### Prevalence of food insecurity

The food insecurity portion of the survey tool was completed by 192 study participants. Approximately 27% of respondents reported being food insecure in the past year (Table 1). Specifically, 72.9% were categorized as high/marginal food security, 21.9% as low food security, and 5.2% as very low food security. In bivariate analyses, there was a statistically significant difference between being food secure and insecure in terms of household income and health insurance status (Table 1).

The chi-square goodness of fit test indicated a statistically significant difference between food insecurity in this study population (27.1%) and that reported by “Asians Only” (1.6%) in the 2016 Clark County CPS (*χ*^2^=31.91, *p*<0.001).

### Univariate logistic regression models

In univariate regression models (Table 2), annual household income, educational attainment, type of health insurance coverage, and type of food usually eaten were significantly associated with food insecurity status. The association between food insecurity and annual household income and educational attainment showed a dose–response relationship. Compared to the highest income group (>$40,000), the middle ($20,000–$40,000) and lower income group (below $20,000) had 2.80 and 4.58 times higher odds of being food insecure. Likewise, compared to those with a graduate degree, participants with lower educational attainment (odds ratio [OR]=1.37 for college degree and OR=2.66 for high school or below) had higher odds of being food insecure, but findings were statistically nonsignificant for college level (*p*=0.454). Compared to the respondents possessing private health insurance, uninsured respondents were 4.5 times more likely to be food insecure. Similarly, those usually eating American or Western food were nearly 3.6 times more likely to report food insecurity compared to those who usually ate Filipino food. See Table 2 for full results.

**Table 2.** Univariate and Multivariable Binary Logistic Regression Results for Factors Associated with Food Insecurity Status in a Subsample of Filipino Americans in Clark County, Nevada

	Univariate		Model 1^a^		Model 2^b^	
	OR (95% CI)	*p*	OR (95% CI)	*p*	OR (95% CI)	*p*
Age	0.99 (0.97–1.01)	0.333	—	—	1.02 (0.97–1.07)	0.438
Highest educational level attained						
Graduate degree or above—Ref.	Reference		Reference		Reference	
College or associate degree	1.37 (0.60–3.10)	0.454	1.14 (0.44–2.93)	0.790	1.94 (0.50–7.53)	0.341
High school or below	**2.67 (1.01–7.02)**	**0.047**	1.25 (0.35–4.42)	0.735	1.72 (0.25–11.66)	0.581
Employment status						
Employed—Ref.	Reference		Reference		Reference	
Unemployed	1.96 (0.74–5.20)	0.179	—	—	5.44 (0.91–32.46)	0.063
Retired	1.26 (0.60–2.61)	0.544	—	—	1.66 (0.33–8.38)	0.538
Annual household income						
Above $40,000—Ref.	Reference		Reference		Reference	
Less than $20,000	**4.58 (2.00–10.51)**	**<0.001**	**4.12 (1.43–11.86)**	**0.009**	**7.19 (1.75–29.55)**	**0.006**
$20,000–$40,000	**2.80 (1.22–6.41)**	**0.015**	1.83 (0.70–4.73)	0.216	1.00 (0.23–4.28)	0.994
Households with children under 18	0.60 (0.25–1.41)	0.240	—	—	2.98 (0.68–13.09)	0.148
Residence by city boundaries						
Henderson—Ref.	Reference		Reference		Reference	
City of Las Vegas	0.62 (0.21–1.84)	0.383	—	—	1.26 (0.22–7.06)	0.795
Clark County	1.19 (0.45–3.14)	0.730	—	—	2.28 (0.50–10.49)	0.289
North Las Vegas	0.57 (0.10–3.33)	0.534	—	—	0.90 (0.06–14.45)	0.943
Resided in the U.S.						
Resided in U.S. for 25 or more years	Reference		Reference		Reference	0.086
Resided in U.S. for 0–10 years	1.07 (0.43–2.70)	0.879			0.61 (0.12–3.08)	0.552
Resided in U.S. for 11–25 years	1.65 (0.79–3.45)	0.184			3.38 (0.82–14.02)	0.093
Usual type of food eaten						
Filipino—Ref.	Reference		Reference			
Western/American	**3.56 (1.04–12.14)**	**0.043**	**7.30 (1.72–30.91)**	**0.007**	**102.60 (8.65–1216.92)**	**<0.001**
Both	1.19 (0.51–2.74)	0.691	1.83 (0.65–5.14)	0.253	**5.34 (1.13–25.15)**	**0.034**
BMI	0.99 (0.93–1.04)	0.566	—	—	1.00 (0.93–1.08)	0.982
Self-reported diabetes (Ref.=no)	1.67 (0.80–3.48)	0.172	—	—	2.58 (0.68–9.79)	0.165
Self-reported hypertension (Ref.=no)	1.47 (0.78–2.79)	0.236	—	—	3.25 (0.74–14.33)	0.120
Self-reported high cholesterol (Ref.=no)	0.76 (0.40–1.46)	0.413	—	—	0.70 (0.20–2.46)	0.577
Type of health insurance coverage						
Private insurance—Ref.	Reference		Reference		Reference	
Public insurance	1.63 (0.80–3.34)	0.179	1.42 (0.62–3.25)	0.405	1.09 (0.26–4.50)	0.908
No insurance	**4.80 (1.82–12.69)**	**0.002**	**5.22 (1.67–16.34)**	**0.005**	**8.84 (1.69–46.29)**	**0.010**

^a^ Model 1 adjusted for significant variables in the univariate analyses (annual household income, highest education level attained, type of food usually eaten, and type of health insurance coverage).

^b^ Model 2 adjusted for all variables of interest listed in Table 2.

Bold values indicate statistical significance, *p*≤0.05.

CI, confidence interval; OR, odds ratio.

### Multivariate logistic regression models

The first multivariable model was adjusted for the four variables found to be significant independent predictors of food insecurity in the univariate analyses (annual household income, highest education level attained, type of food usually eaten, and type of health insurance coverage). Higher odds of being food insecure for the uninsured, low-income participants, and those usually consuming Western or American food were retained in this model after adjustments, but educational attainment lost statistical significance. See Table 2 for full results.

The second multivariable model included all variables of interest regardless of their significance in the univariate analyses. Findings were similar to the first multivariable model and two variables, usually eating both Western/American and Filipino food and currently being unemployed, gained statistical significance and were associated with higher odds of being food insecure. See Table 2 for full results.

## Discussion

This study found that a sizeable proportion of Filipino American study participants reported being food insecure, which was significantly higher than the reported “Asian Only” food insecurity rates from the Clark County Nevada CPS.^19^ This discrepancy in food insecurity rates indicate the heterogeneity among Asian subpopulations. Thus, our study provides evidence in support of previous calls to disaggregate data and recognize the heterogeneity inherent to the Asian American community.^9,10^

The intrapersonal (household income below $20,000 and eating American-Western food) and policy (no health insurance) levels of the Social Ecological Model accounted for significant variance in predicting food insecurity status among the sample population. The increased ORs among variables in the multivariate model compared to the individual univariate models suggests a potential additive effect between the intrapersonal and policy levels. As the Social Ecological Model posits, the different theoretical levels interact in a dynamic and reciprocal manner to influence the target health behavior or outcome.^21^ Thus, the model assumes the individuals and their environment work in an interdependent manner.

Our findings are similar to other studies^2,25,26^ in that annual household income significantly predicted food insecurity. A stepwise gradient existed between food security and income, in which higher odds of food insecurity was observed as the classified income brackets decreased. However, the middle-income category ($20,000–$40,000) lost statistical significance in the multivariable model, which may be due to small sample size or may be the consequence of the interaction between the sociodemographic variables, which in turn is indicative of the complex nature of food insecurity. Given that socioeconomic disadvantage is a strong predictor of food insecurity, policy makers, researchers, and Filipino-based community resource workers should be cognizant of this connection.

Usually eating Western/American food, which served as a proxy for acculturation status, was the strongest predictor of food insecurity in the multivariable models. Acculturation is commonly defined as “the process by which a group, usually a minority group, adopts the cultural patterns of a dominant or host group.”^27^ Thus, usually eating Filipino foods was assumed to indicate less acculturation. Acculturation and its relationship to food security status have not been extensively studied in Filipino American populations or Asian Americans in general. Previous findings on ethnic minorities and immigrant populations and food insecurity have been mixed,^28–30^ thus implicating the complexity of this relationship. Future studies seeking to expand upon food insecurity and acculturation in Filipino Americans should utilize a longitudinal study design and a validated Filipino American-specific acculturation tool rather than proxy measures.^31^ Key informant interviews and focus group sessions may add insight into acculturation patterns adopted by food insecure Filipino Americans.

Similar to pervious findings,^4,25^ a lack of health insurance coverage, which represented the policy level, was a significant predictor of food insecurity. The reciprocal nature between competing financial demands could explain this connection. Further, this relationship may be considered as a vicious cycle, in which food insecure individuals manage by reducing medication to afford food, and likewise, going hungry to afford medication. With the recent efforts to repeal the Affordable Care Act, this field of research should gain increased attention. Filipino American community advocates and leaders should seek to support legislative provisions that expand or provide health insurance coverage to the uninsured, as it has the potential to effect food insecure-experiences.

Researchers have acknowledged the need to conduct studies that investigate Asian American health-related outcomes within the context of social ecological models.^8^ The Social Ecological Model posits that changes among the macrolevel determinants to health will ultimately produce changes at the individual level.^21^ As such, multilevel health promotion efforts that focus on factors at all levels of the model are necessary to affect health outcomes and health behaviors.^21^ This study provides evidence that factors on multiple levels play a role in food security status among Filipino Americans. Overall, Filipino American community advocates need to consider multilevel, intercollaborative interventions when attempting to mitigate food insecurity among Filipino Americans in Clark County.

Statistically significant results were not achieved for many variables of interest, both at the interpersonal level and community level; for example, households with children younger than the age of 18 years are more likely to report experiencing episodes of food insecurity.^2,26^ The lack of statistical significance may be due to small sample size, and thus, results from prior studies should not be ignored. Future research should continue to examine food insecurity in the Filipino American community through the lens of the Social Ecological Model and work toward building a complete picture of food insecurity.

Methodological issues such as sampling bias, a lack of disaggregated data, inconsistent/nonstandardized definitions for Asian Americans, uneven distribution of geographic representation, and small sample sizes in large national surveys have been identified as problems in the collection and reporting of Asian American data,^6,10^ which may have impacted, specifically underestimated, the food insecurity estimates from the CPS. Furthermore, according to 2007–2009 American Community Survey 3-Year Estimates,^32^ one-third of Asian Americans identified themselves as limited English proficiency (LEP) and over one-quarter of Asian Americans in Las Vegas are LEP,^9^ which may lead to sampling bias as , and linguistically isolated individuals are more likely to be of lower socioeconomic status, more likely to either refuse participation or be considered ineligible to participate by the surveyor, or may not have the ability to sufficiently answer survey questions.^6^ Prominent Asian American researchers posit that cultural attitudes toward the aversion of survey participation also reduce response rates in Asian subpopulations.^7^ Thus, studies conducted among Asian Americans contain higher numbers of English-speaking, well-acculturated, more educated, and high-income respondents.^33^ As evidenced by this study, disaggregation of the food insecurity data among Asian Americans is necessary to accurately monitor food insecurity prevalence rates. National surveys such as the CPS may leverage these findings to establish a more effective protocol to sample Asian American subpopulations, including Filipino Americans, to more accurately reflect the differences in social and health-related outcomes among Asian American subgroups.

### Study limitations

The results from this study should be viewed in light of its limitations. The cross-sectional study design does not allow for the establishment of a temporal or causal association. Convenience sampling limits the external validity or generalizability of the findings. Relatedly, the large confidence intervals reported for some of the ORs, likely a consequence of small sample size, reduced the precision of the results. All variables were self-reported and are thus susceptible to potential recall bias and social desirability bias. Despite the limitations inherent to self-reported surveys, this method is regarded as a feasible and effective means of collecting actionable information for research purposes among a large population.^34^ Finally, while the 18-item Household Food Security Survey Module (HFSSM) has been found to be a valid and reliable instrument for assessing food insecurity in Asian and Pacific Islander populations,^35^ three items contained within the HFSSM displayed problematic response rates among Asian and Pacific Islanders residing in Hawaii.^35^ As those three items are also contained within the Short Form Food Security Survey Module, it may present potential issues with regards to the validity and utility of these measures.^24^ However, it is unclear if Asians and Pacific Islanders residing in different geographic regions of the U.S. would also have problematic response rates.^35^

## Conclusions

Overall, 27.1% of Filipino American respondents reported experiencing food insecurity at some point during the year, with 5.1% of participants reporting very low food security. Factors at various levels of the Social Ecological Model, education, household income, type of food usually eaten, and health insurance status were associated with food insecurity. These findings are an essential first step to understanding the breadth of factors that influence food insecurity in this unique Asian subgroup. Consequently, this study's findings hold major implications for local and national Filipino American community leaders and advocates and subsequently can be translated into tangible prevention and support programs. Targeted interventions and health promotion programs should be cognizant of unique cultural and demographic characteristics of Asian American subgroups and use multilevel, intercollaborative efforts targeted at reducing food insecurity.

## Abbreviations Used

BMI: body mass index
CI: confidence interval
CPS: Current Population Survey
HFSSM: Household Food Security Survey Module
LEP: limited English proficiency
OR: odds ratio
SD: standard deviation
U.S.: United States
USDA: United States Department of Agriculture
